# A Novel Part Refinement Tandem Transformer for Human–Object Interaction Detection

**DOI:** 10.3390/s24134278

**Published:** 2024-07-01

**Authors:** Zhan Su, Hongzhe Yang

**Affiliations:** School of Computer Science and Technology, Dalian University of Technology, Dalian 116024, China; hongzheyang_6163@sina.com

**Keywords:** HOI detection, deep learning, vision transformer, visual relationship

## Abstract

Human–object interaction (HOI) detection identifies a “set of interactions” in an image involving the recognition of interacting instances and the classification of interaction categories. The complexity and variety of image content make this task challenging. Recently, the Transformer has been applied in computer vision and received attention in the HOI detection task. Therefore, this paper proposes a novel Part Refinement Tandem Transformer (PRTT) for HOI detection. Unlike the previous Transformer-based HOI method, PRTT utilizes multiple decoders to split and process rich elements of HOI prediction and introduces a new part state feature extraction (PSFE) module to help improve the final interaction category classification. We adopt a novel prior feature integrated cross-attention (PFIC) to utilize the fine-grained partial state semantic and appearance feature output obtained by the PSFE module to guide queries. We validate our method on two public datasets, V-COCO and HICO-DET. Compared to state-of-the-art models, the performance of detecting human–object interaction is significantly improved by the PRTT.

## 1. Introduction

Given an image containing multiple humans and objects, human–object interaction (HOI) detection is a task of predicting a set of ⟨human, object, interaction⟩ triples in an image [1]. Due to its grand prospect in high-level human-centric scene understanding tasks, precise detection of human–object interactions can profit numerous subsequent activities, including action detection [2,3] and scene graph generation [4,5], so it has recently attracted considerable research interest.

Traditional HOI detection methods are generalized into two modes: two-phase or one-phase. In the basic two-phase detection framework, human and object features are often extracted using an object detection network, from which interactions are inferred. For the two-phase mode, many researchers use additional features such as relative spatial configuration [6,7], interactiveness field [8], human pose estimation [9], body part features [10], or scene graph [11] to enhance these features. However, in the object detection stage, the exhaustion of human and object instance pairings and some post-processing, such as NMS, lead to additional time complexity. For faster detection, the single-stage mode performs interaction prediction and object detection in parallel [12,13]. However, the detection result will be inaccurate when the image’s content is a multi-overlapping crowd scene or a special scene, such as humans and objects far away in space.

Recently, Vision Transformer [14] has revolutionized tasks in the vision domain, overcoming traditional methods’ problems and implementing a competitive technique in both accuracy and detection time for HOI detection. This article belongs to Transformer-based work. The Transformer architecture’s self-attention and cross-attention [15] can better obtain the contextual information between different instances, which is particularly appropriate for detecting HOI. QPIC [16] and HOITransformer [14] define a set of learnable queries containing different types of elements to compose HOI triplet predictions. HOTR [17] sets two sets of queries for a pair of parallel decoders, but it uses a complex pointer mechanism to combine the outputs of the two tasks. Similarly, HOICLIP [18] uses matching to obtain the initial query group for interactive classification. CDN [19] adopts a dual decoder design, but it only uses the previous query result as the input of the next query with a simple guidance strategy. Zhang et al. [20] exploited unary and pairwise representations for HOIs with the same Transformer. Most works follow the simple design of utilizing a single decoder to predict all HOI prediction elements directly. Although this architecture is successful, it also has its drawbacks: (i) Due to the ambiguity of interactions in special cases (for example, when a human stands in front of a motorcycle, the interaction between the human and the motorcycle is most likely to occur in image space, but the human is not riding a motorcycle) and the large gap from pixels to activity concepts, it is not enough to find contextual features by simple initialized query, and self-attention mechanisms in one-shot networks. Interaction queries require additional guidance. (ii) Since the HOI prediction contains too many elements (human location, object location, instance category, and interaction category), it is not easy to focus on all element-related features and achieve a good trade-off using only a set of simple queries. However, in some works [14,21], multiple queries require additional and time-consuming matching operations. Our work addresses these issues well.

**Figure 1:**
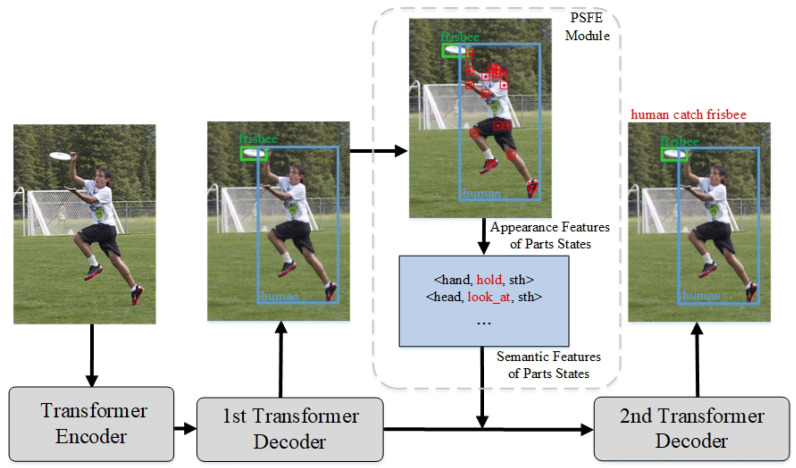
We equip the same encoder with multiple tandem decoders to handle the HOI prediction composed of human–object (HO) pair recognition and interaction category detection, respectively. In the intermediate stage, we utilize the PSFE module to extract appearance features of part states based on human pose key points and further generate semantic features of part states to refine the representation of queries in the second stage.

We propose a novel end-to-end model, Part Refinement Tandem Transformer for HOI Detection (PRTT), to address the above drawbacks. It introduces a part state feature extraction (PSFE) module to improve the previous Transformer-based HOI detection design in the intermediate stage. The local features between human body parts and objects are essential in interactions. For instance, when a photographer operates a camera, it is crucial to analyze how the photographer’s hands interact with the camera. The positioning, posture, and contact points between the hands and the camera deliver key insights into the mechanics, timing, and reasons behind the interaction. By leveraging these features as guiding elements, we can significantly bridge the gap from mere pixels to meaningful activity concepts, thus providing more profound and accurate contextual guidance for detecting human–object interactions (HOIs). The main innovative idea of PRTT is shown in Figure 1. It utilizes human pose key points as clues to extract the appearance features and semantics features of human part states and encodes them to support and guide queries. Simultaneously, for the second drawback, PRTT effectively focuses on all element-related features and achieves a good trade-off by disassembling and querying the rich HOI prediction elements multiple times. Through the multiple tandem decoders strategy, the output of the previous decoder query is utilized as the input of the next decoder query. The two query results correspond individually, avoiding the additional and time-consuming matching operations [14,21]. Lastly, experiments on the HICO-DET [1] and V-COCO [22] datasets demonstrate the effectiveness of our method. The main contributions of our paper can be summarized as follows:

- This study disassembles the rich HOI prediction elements and performs multiple queries to focus on all element-related features and achieve a good trade-off effectively. Simultaneously, it adopts the multiple tandem decoders strategy to avoid additional and time-consuming matching operations.
- We efficiently encode and integrate appearance features and state semantics through a pretrained Bert model with human pose key points as clues.
- This study adopts a novel prior feature integrated cross-attention layer to efficiently introduce fine-grained part-state semantics and appearance features in the second stage to guide and improve queries.

## 2. Related Work

### 2.1. Object Detection

Object detection based on CNN is divided into two-stage and one-stage object detection according to its structure. Girshick et al. [23] proposed R-CNN, which has also become the originator of CNN-based object detection. Girshick et al. [24] proposed Fast R-CNN based on R-CNN, using RoI Pooling Layer, adding the classification step and bounding box regression after feature extraction. Compared with the multi-stage training of R-CNN, the training of Fast R-CNN is more concise and efficient. He et al. [25] proposed a Mask R-CNN model for image segmentation task, adding a parallel branch for predicting object masks based on Faster R-CNN. Redmon et al. [26] proposed an object detection model named “You Only Look Once” with only one forward pass. At the beginning of 2018, YOLOv3 underwent major changes in the overall structure compared with the previous one, using multiple independent logical classifiers to replace the softmax function [27], and it has developed into YOLOv8 by 2023 [28]. DETR (Detection Transformer) [21] first used a transformer in computer vision to implement an end-to-end object detection method. The results are comparable to Faster R-CNN on the COCO small object dataset but outperform Faster R-CNN on large objects. Numerous approaches to improving DETR performance have subsequently emerged.

### 2.2. Human–Object Interaction Detection

Many traditional HOI detection methods using CNN features have promoted the advancement of HOI detection, which is often divided into two paradigms: two-phase strategy and one-phase strategy.

Two-phase strategy: In the first stage, off-the-shelf object detectors are usually utilized to localize objects, including humans and objects. Interaction labels are then predicted in the second stage by incorporating additional features. Chao et al. [1] proposed a three-branch network, HO-RCNN, to extract the features of human–object spatial relations. Gao et al. [7] proposed a human-centric attention module, iCAN, to emphasize important regions related to interactions in the image. Additionally, other works fuse pose estimation with visual features to provide accurate features of the human form, further improving network performance. Li et al. [29] used the pose estimation network and the human pose stream to extract human pose features. Many works [10,30,31] have involved body part features as important auxiliary features in interaction detection. Liu et al. [32] constructed a body-part-based dataset, HAKE, and proposed a multi-level pairwise feature network, PFNet. However, two-phase methods are usually inefficient due to handling many noninteractive detected objects and redundant combinations of human object instances, whereas the accuracy of object detection greatly influences the network’s performance.

One-phase strategy: Recent works have attempted to address the problems faced by two-phase networks within a single-phase framework and have attracted widespread attention. Based on CenterNet [33], Liao et al. [12] proposed PPDM (parallel point detection and matching), where the point-matching branch matches human and object points originating from the same interaction point. Such operations reduce the number of candidate interaction points screened and save computational costs. IP-Net [13] is similar. Zhong et al. [34] designed a single-stage GGNet (glance and gaze network) to adaptively model a set of action-aware points in two steps of glance and gaze to improve the performance of point-based policies. UnionDet [35] eliminates the extra inference stage by directly predicting the union box with an extra branch. Despite the great efficiency gains, this strategy combines two different tasks and poses a great limitation in terms of performance.

Recently, a Transformer architecture [36] was applied in various tasks in computer vision, such as video inpainting [37] and medical image quality assessment [38], and it achieved state-of-the-art performance. Transformer-based one-phase methods treat it as a set prediction problem in the HOI detection task. More specifically, following DETR [21], HOI-Transformer [14] and QPIC [16] use a typical transformer with encoder–decoder architecture to define the prediction of a learnable query as <human, object, action> triples. In these works, matching instances before interactive classification is unnecessary. HOTR [17] and AS-Net [39] deploy dual concurrent decoders for predicting HO pairs and interaction classification correspondingly and then perform a matching operation on the output results of the two decoders. Although these transformer-based methods achieve remarkable performance, these studies depend solely on self-attention mechanisms for discovering prominent context features, where queries during interaction classification are initialized to zero. This results in a lack of corresponding guidance in the query process and the subsequent need for a time-consuming matching process. Instead, we introduce the semantic features of part states as additional features and input them into PFIC layers together with the output of object detection to guide queries for interactive category classification. There is a one-to-one correspondence between the outputs of the tandem structure, which saves the matching operation process.

## 3. Method

### 3.1. Overview

**Figure 2:**
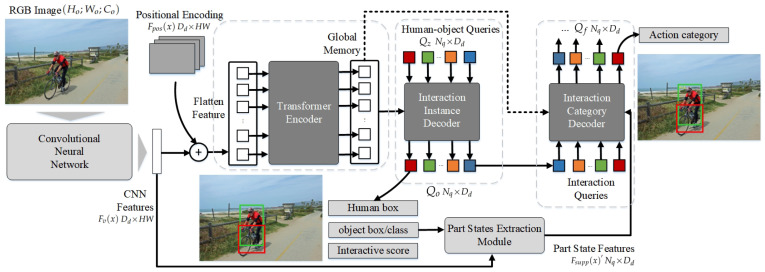
Overview of PRTT: Interaction Instance Decoder and Interaction Category Decoder are run in tandem, sharing the same Transformer encoder. In the intermediate stage, we utilize the PSFE module to generate N vectors representing semantic and appearance features of part states to guide the queries in the next stage. Then, the interaction and HO pair representations are obtained separately in the two concatenated decoders and combined into HOI triples. Here, ⊕ represents concatenation procedures.

Figure 2 shows the specific implementation process framework of the idea in Figure 1. Our proposed PRTT consists of four steps. Following the processing steps of DETR [21], we extract its features using a CNN backbone and combine positional encoding inputs to a Transformer encoder to transform these. Then, a set of queries is input into the Interaction Instance Decoder to identify the HO pair instance proposals and interaction scores. Next, the feature map extracted by CNN and the proposals obtained in the previous step are input into the part state feature extraction module to obtain *N* part state features. After that, we utilize the output of the previous decoder, *N* partial state features, and global memory as the input of the Interaction Category Decoder to query the interaction category corresponding to the HO pair. Finally, we combine the outputs of the two tasks to form HOI triples.

### 3.2. Backbone

The input is an RGB image of shape $\left(H_{o};W_{o};C_{o}\right)$, where $H_{o}$, $W_{o}$, and $C_{o}$ represent the picture’s dimensions and RGB channels. We use a standard CNN feature extractor network to obtain feature maps indicated for $F\left(x\right)\in R^{H\times W\times D_{c}}$. Subsequently, we feed F(x) into a layer of convolution using a kernel size of 1×1, which reduces its channel dimension $D_{c}$ to a smaller value $D_{d}$. The new feature map is $F\left(x\right)\in R^{H\times W\times D_{d}}$, where $D_{d}$ defaults to 256. Next, we flatten $F_{d}\left(x\right)$ using the flatten operator to generate the flatten feature $F_{v}\left(x\right)\in R^{D_{d}\times HW}$. Following previous work [14,16,17,39], we add a fixed positional encoding $F_{pos}\left(x\right)\in R^{D_{d}\times HW}$ in the $F_{v}\left(x\right)$ to add the position details. The encoder implements the regular structure of transformers and is composed of several encoder layers, in which each of them is primarily composed of a self-attention layer and a feedforward (FFN) layer. It aggregates global information to output a global memory of dimension $D_{d}$. The calculation process of the transformer encoder is as follows:

(1) $$F_{enc}\left(x\right)=f_{encoder}\left(F_{v}\left(x\right),F_{pos}\left(x\right)\right)$$

### 3.3. Tandem Transformer

Multiple tandem decoders strategy: In our proposed tandem transformer architecture, the HOI predictions are divided into HO pair recognition and interacting category identification, similar to the two-phase structure method. The two-phase approach utilizes the off-the-shelf object recognizer for preprocessing, while the subsequent network focuses on interaction category classification using the feature maps obtained from the recognizer. This design allows the two-phase HOI detection method to perform better and be more stable than traditional single-stage detection methods. Therefore, the two decoders can focus on corresponding task-related features from the global memory shared by the encoder output. The multiple tandem decoders strategy enables the query process of the two decoders to correspond one to one, which enables the output results of the two queries to be directly combined to form HOI predictions.

Interaction Instance Decoder: The Interaction Instance Decoder we designed refers to the basic structure of the transformer-based object detector DETR [21]. It is composed of several standard transformer decoder layers, each containing a self-attention component, FFN, and a cross-attention layer. The cross-attention layer aggregates the embedding features $F_{enc}\left(x\right)$ output by the encoder into $N_{q}$ queries. We take the learnable query $Q_{z}\in R^{N_{q}\times D_{d}}$ and the encoder output global memory as input. It is transformed into another set of output queries $Q_{o}\in R^{N_{q}\times D_{d}}$. For each query, PRTT applies three FFN heads and one binary score head to predict human bounding boxes, object bounding boxes, object categories, and binary interaction scores, thus composing the set of interaction instances pair predictions $P_{o}$ and the corresponding interaction scores. The interaction score (IS) indicates whether the interaction instance pair produces an interaction. For samples without interaction, it has the effect of reducing the final score. Therefore, the Interaction Instance Decoder can be expressed as follows:

(2) $$P_{o}=f_{decoder_{z}}\left(F_{enc}\left(x\right),Q_{z},F_{pos}\left(x\right)\right)$$

We send output queries $Q_{o}$ to the interactive category decoder. Simultaneously, PRTT performs the part state feature extraction on the prediction $P_{o}$ of the Interactive Instance Decoder and the feature map of the CNN backbone. Here, we first perform pose estimation on the human region in the image to obtain *N* key points according to a set of HO pair predictions of the previous decoder. Next, PRTT crops the *N* body part area features and the object region features on the CNN feature map. The combinations of these features are input to our PSFE (part state feature extraction) module. Then, we utilize the human body part state semantic clues to generate the part state vector as the supporting feature $F_{supp}\left(x\right)$. The implementation describes for the PSFE module are provided in Section 3.4. Therefore, obtaining the supporting feature $F_{supp}\left(x\right)$ can be simply represented as follows:

(3) $$F_{supp}\left(x\right)=f_{supp}\left(F\left(x\right),P_{o}\right)$$

**Figure 3:**
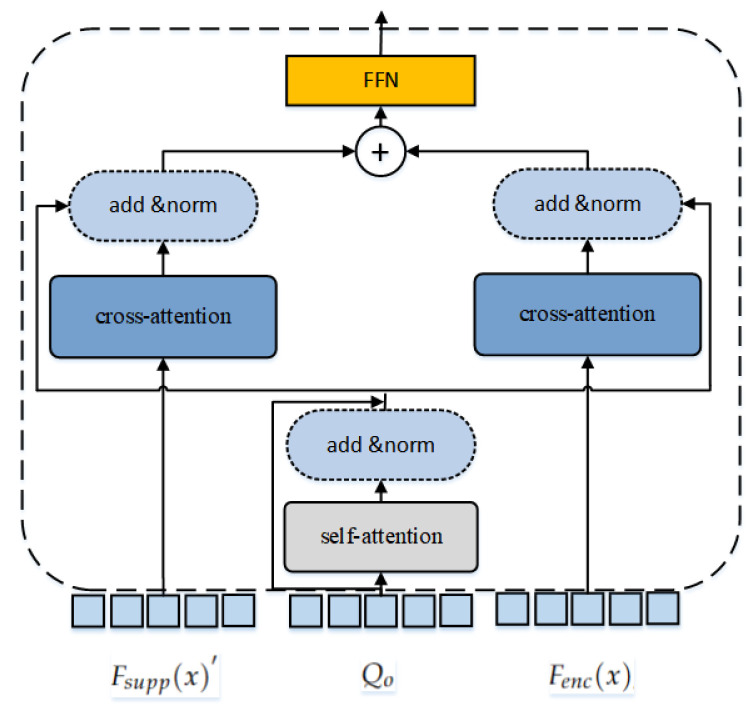
Structure of prior feature-integrated cross-attention layer.

Interaction Category Decoder: We utilize the Interaction Category Decoder to classify the corresponding interaction instance pairs, a multi-label classification task. To better guide it to aggregate classification-related features, we utilize supporting features $F_{supp}\left(x\right)$, a set of output queries $Q_{o}$ and global memory as input to the Interaction Category Decoder. In this process, we project the supporting features $F_{supp}\left(x\right)$ to the same feature space as $Q_{o}$ to obtain $F_{supp}{\left(x\right)}^{\prime }$. The Interaction Category Decoder consists of *M* PFIC layers, and its structure is shown in Figure 3. $Q_{o}$ is processed through multiple PFIC decoder layers and converted into another set of output queries $Q_{f}$. After passing through the FFN header, a collection of interacting classes $P_{f}=\left\{a_{i}|i\in \left\{1,2,...,N_{d}\right\}\right\}$ is generated. Thus, the Interaction Category Decoder can be represented as follows:

(4) $$P_{f}=f_{decoder_{o}}\left(F_{supp}{\left(x\right)}^{\prime },Q_{o},F_{enc}\left(x\right),F_{pos}\left(x\right)\right)$$

In the PFIC layer, $Q_{o}$ is first updated by self-attention and then input to the cross-attention modules of $F_{enc}\left(x\right)$ and $F_{supp}{\left(x\right)}^{\prime }$ respectively. The two output features are obtained. The two outputs are then added and fed into a feedforward network, as shown in the following formula:

(5) $$\begin{array}{cc} \;Q_{f} & =SelfAttn(Q_{f}) \end{array}$$

(6) $$\begin{array}{cc} \;C_{f} & =CrossAttn(Q_{f},F_{enc}\left(x\right)) \end{array}$$

(7) $$\begin{array}{cc} \;S_{f} & =CrossAttn(Q_{f},F_{supp}{\left(x\right)}^{\prime }) \end{array}$$

(8) $$\begin{array}{cc} \;Q_{f} & =FFN(C_{f}+S_{f}) \end{array}$$

### 3.4. Part State Feature Extraction

**Figure 4:**
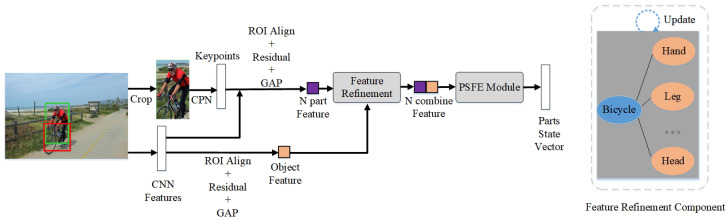
Illustration of the process of part state feature extraction. Here, GAP is global average pooling, and Residual denotes residual block.

Based on the CNN feature map and the prediction of the Interaction Instance Decoder, PRTT obtains each part state feature (appearance visual feature and semantic feature) of a human in the HO pair as additional features. The process is shown in Figure 4. Firstly, we crop the human region according to the prediction result of the Interaction Instance Decoder and utilize CPN [40] to perform the pose estimation operation on it to obtain the coordinates of *N* key points $L_{pn}=\left\{x_{pn},y_{pn}\right\}$. Then, with *N* key points as the center, a region $R_{pn}=\left\{h_{pn},w_{pn},x_{pn},y_{pn}\right\}$ is generated, where $h_{pn}$ and $w_{pn}$ are the height and width of the square region of the human part. The following is the calculation formula:

(9) $$h_{pn}=w_{pn}=\left[\gamma \sqrt{S_{human}}\right]\;1\leq n\leq N$$

where $S_{human}$ represents the area of the human region, the [] denotes a rounding operation, and γ denotes a scale parameter empirically determined to be 0.1. Thus, we have N+1 regions of human parts and the object. Then, we perform ROI Align, residual and GAP operations on the F(x) to produce N+1 region features.

Feature refinement: Referring to the work in PGPN [30], the features are propagated through one GCN layer according to the graph structure in the Feature Refinement Component in Figure 4. The refined part features collect the feature information of the object, which is equivalent to using the advanced features of the object. By avoiding the repetition of human and parts information, this refinement process can increase interaction detection accuracy. We connect *N* refined part features ${\left\{f_{pn}\right\}}_{n=1}^{N}$ and refined object features $f_{object}$ to form N combine feature vectors and input them into the PSFE module to calculate the parts state vectors.

**Figure 5:**
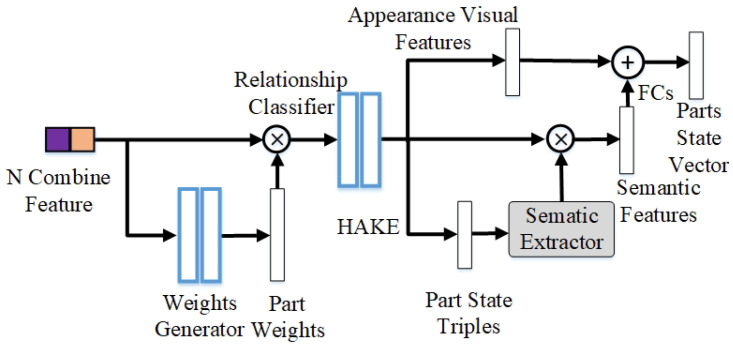
Illustration of the process of the PSFE module. Here, ⨂ represents element-wise multiplication, ⊕ is the concatenation process, FCs denote two fully connected layers, and the semantic extractor is a BERT-based pretrained model.

As presented in Figure 5, the *N* combined features will first be input to the weight generator. The weight of a human part represents the importance of the part to the interactions between the corresponding HO pair instances. For example, the state of the head has little effect on judging the interaction of the “ride bicycle” label. On the contrary, the state of the head is very important for the interaction of the “talk on a phone” label. This section refers to the related work of HAKE-Action [32], and our weight labels are directly converted from the labels in the HAKE dataset. If the label of the part and the corresponding object in HAKE is “no interaction”, its value is set to 0; in contrast, the body part has clues to the inference of interactions, and its value is set to 1. With weight labels as supervision, we utilize a weight generator consisting of fully connected layers and sigmoids to generate a set of weights ${\left\{\alpha_{n}\right\}}_{n=1}^{N}$ for every HO pair. Therefore, the calculation process for weights among the human part–object can be expressed as follows:

(10) $$\alpha_{n}=f_{weight}\left(f_{pn},f_{object}\right)$$

We multiply the original combined feature with weight to obtain a new feature $f_{pn}^{*}$. After that, relationship recognition is performed for each human part–object pair, and its output is a triple in the form of <part, verb, object>. Relationship recognition is a multi-label classification task, such as <hand, hold, ball>, and <hand, throw, ball> may be correct simultaneously, so we utilize a relationship classifier consisting of multiple fully connected layers and multi-sigmoids to deal with it. The loss used in generating weight and relationship recognition is as follows:

(11) $$L_{ps}=\sum_{n}^{N}\left(L_{weight}^{n}+L_{r}^{n}\right)$$

where $L_{weight}$ is the cross-entropy loss that generates weight $\alpha_{n}$, and $L_{r}$ is the cross-entropy loss of relationship recognition. We obtain a relational triplet set of N human part–object pairs from this. From this set of triples, we innovatively extract the corresponding semantic feature ($PSFE_{s}$) through the BERT-based pretrained model [41]. Specifically, the human part–object contains *m* relational triples, and each word in each relational triple acquires a 768-dimensional feature through the pretrained Bert model. Then, PRTT performs the concatenate operation and multiplies the corresponding probabilities score of its triple in the relationship classifier as the semantic feature of the 2304*m-dimension of this part. After that, we utilize the pool and resize operations to reduce it to 3584 dimensions. Finally, we concatenate the semantic feature with the 512-dimension appearance visual feature ($PSFE_{v}$) extracted directly from the last FC layer of the relationship classifier to obtain 4096-dimensional additional features $f_{ps}$.

### 3.5. Inference and Loss Function

The loss calculation consists of two steps: a bipartite matching step between predictions and ground truth and a loss calculation step for matched pairs. For bipartite matching, we fill the ground truth set of HO pairs with φ (no pair), expanding the ground truth set to $N_{q}$. Our work follows the training process of QPIC [16] and matches each ground truth with its best matching prediction using the binary matching of the Hungarian algorithm [42]. A loss is generated between the matched predictions and the corresponding ground truths. Here, the prediction contains the output of two tandem decoders. In addition to following QPIC’s box-regression loss $L_{b}$, intersection-over-union (IoU) loss $L_{u}$, object-class loss $L_{c}$, and action loss $L_{a}$, the PRTT loss also includes the interactive score loss $L_{s}$ corresponding to the interaction score output by the Interaction Instance Decoder.

(12) $$L_{f}=\sum_{k\in (h,o)}\left(\lambda_{b}L_{b_{k}}+\lambda_{u}L_{u_{k}}\right)+\lambda_{c}L_{c}+\lambda_{a}L_{a}+\lambda_{s}L_{s}$$

where $\lambda_{b},\;\lambda_{u},\;\lambda_{c},\;\lambda_{a},\;\mathrm{and}\;\lambda_{s}$ are the hyperparameters for adjusting the weights of each loss.

After tandem decoders generate the output results, since the query output by the Interaction Instance Decoder is refined by the additional feature as the learnable query input of the Interaction Category Decoder, the relationship between the output results is also one-to-one correspondence, so we can combine them to obtain five-tuple <human bounding box, object bounding box, object class, interactive score, interaction class> output set. $<b_{j}^{h},b_{j}^{o},argmax_{k}s_{j}^{hoi}\left(k\right)>$ is the *j*-th prediction. Then, we set the prediction score $s_{j}^{hoi}$ as $s_{j}^{a}s_{j}^{o}s_{j}^{i}$, where $s_{j}^{a}$ and $s_{j}^{o}$ are the scores of interaction classification and object classification, respectively, and $s_{j}^{i}$ is the score of whether the HO pair produces an interactive action. Simultaneously, the prediction scores are also used to sort the prediction set. We adopt the pairwise nonmaximum suppression method [19] to filter the repeated prediction results and take the top *K* HOI prediction results after sorting. In this process, the threshold value, parameter α, and parameter β take 0.7, 1, and 0.5, respectively.

## 4. Experiments

In this section, we summarize our experimental results to demonstrate the superiority of our proposed model. First, Section 4.1 briefly introduces the datasets and experimental metrics we use. Then, Section 4.2 describes the implementation details of our model. We then evaluate the performance of PRTT by comparing it with previous state-of-the-art methods. Finally, in Section 4.5, we perform an ablation study to validate our design choices.

### 4.1. Experimental Setup

Datasets: We evaluate PRTT on two publicly available datasets: V-COCO [22] and HICO-DET [1]. HICO-DET has a total of 47,776 images: 38,118 for training and 9648 for testing. These include 600 HOI categories (full) with over 117 interactions and 80 object categories. Based on the training instances, it is further divided into 138 Rare (HOI categories with less than ten samples) and 462 Non-Rare (HOI categories with more than ten samples). V-COCO is a relatively small dataset, a subset of COCO [43]. It consists of 2533 and 2867 images for training and validation and 4946 for testing. The images are annotated with 80 objects and 29 action classes. Of its 29 classes, four lack object annotations, and one has only 21 photos in its sample pool. In Section 3.4, we refer to the relevant work of HAKE Action and its dataset labels. The HAKE dataset is a knowledge-driven dataset. HAKE includes 26 $M^{+}$ human body component level atomic action labels (component status), logic rules of component status, overall object knowledge labels (category, attribute, affordance), and their causal relationships.

Evaluation metric: The prediction is correct for a positively predicted HOI triplet <human, action, object> if the predicted human and object bounding boxes overlap with their respective ground truth boxes (IoU greater than 0.5) and the predicted interaction class is correct. We follow the protocol recommended by both datasets [1,22] to evaluate the results on both datasets using mean average precision ($mAP_{role}$) [1]. For V-COCO, there are two protocols: Scenario 1 and Scenario 2. When there is interaction without any objects (humans only), Scenario 1 regards a tough evaluation criterion requiring an empty bounding box with origin coordinates. Another scenario settles the situation by skipping the predicted bounding box for evaluation for such cases.

### 4.2. Implementation Details

As the backbone of PRTT, we select the ResNet-101 with a 6-layer transformer encoder as the visual feature extractor based on its performance on the V-COCO and HICO-DET training sets. We deploy the PSFE module to obtain additional features, and this module utilizes CPN to estimate human pose. These are pretrained on the HAKE dataset. During the training phase, we follow the strategy of the previous work [14,16,17,39] to initialize the network with the parameters of DETR [21] pretrained on the MS COCO dataset. The dimension of queries $D_{d}$ is set to 256. For the HICO-DET and V-COCO datasets, $N_{q}$ is set to 64 and 100, respectively. Using the AdamW [44] optimizer with a weight decay of 0.0001, we train the entire model on 100 epochs, set the learning rate to 0.0001 out of the initial 60 epochs, and then reduce it to one-tenth of the original. Our network is trained on GeForce RTX NVIDIA GPUs (8 × 2080Ti) (NVIDIA, Santa Clara, CA, USA) with a batch size of 16. Each decoder in our work is equipped with a 6-layer transformer. Of the two decoders, the FFN header for the output human and object boxes has three linear layers with ReLU, while the FFN header for the output object and interaction category has only one linear layer. For the loss function, we follow the work of QPIC by setting $\lambda_{b},\;\lambda_{u},\;\lambda_{c},\;\lambda_{a}$, and $\lambda_{s}$ to 2.5, 1, 1, 1, and 1, respectively.

### 4.3. Results

In this section, we compare the performance of PRTT with current state-of-the-art methods, as shown in Table 1 and Table 2. As described in Section 4.1, we follow the proposed evaluation protocol on the V-COCO [22] and HICO-DET [1] datasets.

As shown in Table 1, our proposed model outperforms the previous state-of-the-art methods by a large margin on the V-COCO dataset. Specifically, compared with PGPN [30], which also uses pose-guided local feature extraction, and SMPNet [31], which uses a multi-level feature fusion strategy including part features, our method has significant advantages, improving by 15 mAP and 12.4 mAP in Scenario 1, respectively. The two-phase method FCMNet [45] with ResNet-50 as the backbone achieves an $mAP_{role}$ of 53.1 in Scenario 1. Using two parallel decoders to process two tasks, respectively, and then pairing the results with pointers, the HOTR achieves an $mAP_{role}$ of 55.2. Compared with ASNet [39], a two-stage method using a similar strategy to HOTR, which has a performance value of 53.9 mAP, our method obtains an improvement of 11.3 mAP. The GTNet [47] proposed the object semantic-guided model trained with relative spatial configuration, which provides $mAP_{role}$ of 56.2 and $mAP_{role}$ of 60.1 in Scenarios 1 and 2. In the current work, the Transformer-based one-phase method QPIC [16] achieves 58.3 $mAP_{role}$ in the Scenario 1 set. The CDN using a simple guiding strategy achieves 63.9 $mAP_{role}$ and 65.9 $mAP_{role}$ in Scenario 1 and Scenario 2, respectively, while our complete model achieves 65.2 $mAP_{role}$ and 66.8 $mAP_{role}$ top results in the two scenarios, respectively.

The results of each method on the HICO-DET dataset are shown in Table 2. As mentioned above, we adopted the evaluation metrics proposed in the work of Chao et al. [1]. Our proposed model is evaluated with default settings on three HOI categories: full, rare, and non-rare. Specifically, compared to QPIC [16], PRTT achieves a gain of 5.16 $mAP_{role}$ on the full set of the HICO-DET dataset. Our method outperforms the SOTA method, achieving an $mAP_{role}$ of 35.06 on the full set. Compared with PGPN [30] and SMPNet [31], which use pose-guided body features for HOI detection, PRTT achieves improvements of 17.66 mAP and 14.75 mAP, respectively. Compared with HOTR [17] and ASNet [39] using parallel decoders, PRTT improves by 9.96 mAP and 6.19 mAP respectively. Using the same backbone ResNet-50, our model performs slightly worse than OCN with 0.3 mAP on the V-COCO dataset but performs much better than OCN [49], with 3.56 mAP on the HICO-DET dataset with fine-grained interaction labels. This is because the additional features extracted by the PSFE module have a significant guiding effect in distinguishing similar interaction categories.

### 4.4. Qualitative Visualization Comparison

**Figure 6:**
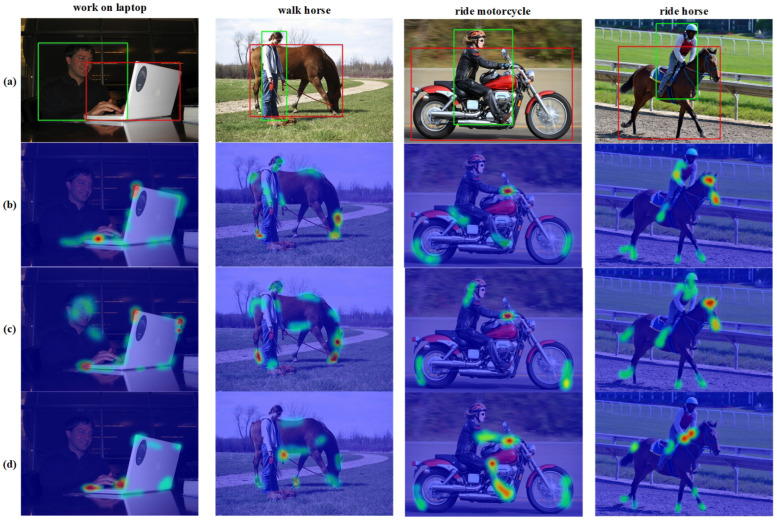
Attention visualization. The attention map is extracted from the decoder’s final layer. In each subgraph, from top to bottom, are (**a**) the original image with ground truth; (**b**) the attention map of QPIC; (**c**) the attention map of the Interaction Instance Decoder; and (**d**) the attention map of the Interaction Category Decoder.

To better analyze the model conduct, we compare the attention maps of the last layer of the decoder from the traditional one-phase method QPIC and the two decoders of PRTT in Figure 6. We can see from the results in the figure that the weights of the Interaction Instance Decoder and QPIC tend to be similar regions (boundaries and intersection regions of the HO pair), so the decoder can effectively collect relevant features to guide the region localization of the HO pair by focusing on these regions. On the other hand, the weights of the Interaction Category Decoder tend to focus on the representative parts and regions of the human to guide the identify the interaction type. Taking the first column in Figure 6 as an example, the two PRTT decoders focus on the laptop’s shape and the hand of the person operating the laptop, respectively. Also, for the rider in the fourth column in Figure 6, the decoder that handles the positioning task focuses more on the edges of the horse and the rider. The decoder dealing with interactive action classification pays more attention to the rider’s bent knees and hand parts because these human parts are used as an additional feature to guide the decoder. Compared to PRTT, QPIC extracts two tendencies of features with a separate decoder, but neither is prominent.

**Figure 7:**
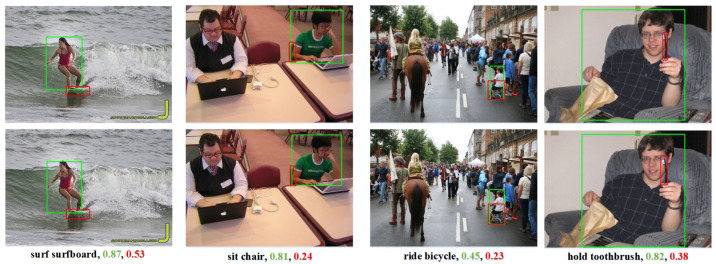
Comparison of the qualitative results of PRTT and QPIC. For the same image, the first row is the prediction results of QPIC, and the second row is the prediction result of the method we proposed. The prediction scores of the two methods are exhibited in the captions. PRTT’s detection scores are labeled with green, and the scores of QPIC are labeled with red. The forecast score is displayed in the captions.

We show the qualitative results of PRTT and compare them to the baseline (QPIC). Figure 7 represents the result of the example chosen from various interaction categories. We find that PRTT produced more reliable scores for HOI, which has an indicative body part. This shows that the part-level feature has effectively guided the decoder to collect more relevant information.

### 4.5. Ablation Study

In this section, we explore how each component of PRTT contributes to the final performance. All experiments are performed on the V-COCO dataset. Table 3 shows the final performance after excluding each component of PRTT in the V-COCO test set. We set a pure model as the base model, which has two tandem transformers to process two tasks separately without additional post-processing operations.

Multiple tandem decoders strategy: As shown in Table 3, we improved the query strategy of two tasks in the same decoder to be processed by two tandem decoders separately, that is, the base model, which achieved 62.1 mAP. There is an improvement of 3.3 mAP compared to the one-phase method QPIC. The result is close to the CDN-B variant’s [19] 62.29 $mAP_{role}$. In this variant, we directly input the output of the previous decoder as a learnable query into the latter decoder without using additional features as guidance, and the Interaction Category Decoder adopts multiple typical transformer decoder layers.

Appearance visual feature ($PSFE_{v}$): This is an important additional feature extracted by the PSFE module before the interactive action classification task: obtaining the appearance features of human body parts from the CNN feature maps and the Interaction Instance Decoder’s prediction. Two simplified versions of our model were executed to evaluate the impact of this component. By comparing the variant model lacking appearance visual feature with the complete model, it is found that FR + $PSFE_{v}$ improves the performance by 0.9 mAP. Compared with the base model, the mAP of the guided query using visual features as additional features in the PFIC layer increases from 62.1 to 63.3 mAP.

Feature refinement (FR): As presented in Table 3, according to the graph structure in Figure 4, PRTT utilizes a GCN layer to update the parts and object features. The refined part features collect the feature information of the object. The repetition of human and component features can be prevented by performing this refinement operation utilizing the object’s high-level features. By comparing the variant model without feature refinement to process the appearance visual feature with the complete model, it is found that FR marginally enhances the model’s effectiveness by 0.3 mAP.

Semantic feature ($PSFE_{s}$): In our model’s PSFE module, we use HAKE labels and BERT to obtain semantic representations of human body part states as additional features to guide the query. A simplified version of our approach is executed without this branch. Compared to other results, experiments display a gain of 1.1 mAP.

Interaction score (IS): This is comparable to the work of Shen et al. [51] but different from QPIC; we add the output $s_{j}^{i}$ value to the Interaction Instance Decoder to measure whether there is an interaction between human and object. It has the effect of lowering the score in the case of no interaction. Performance is improved by 0.4 mAP with IS.

## 5. Conclusions

In this paper, we introduce PRTT, a novel Transformer-based ensemble prediction method proposed for detecting human–object interactions. The model utilizes multiple decoders to split and process the elements of HOI prediction correspondingly to focus on the features related to the elements. In the intermediate stage, we utilize the pretrained Bert model to encode part-state semantic and appearance features to guide and improve queries by the PFIC layer. PRTT exhibits superior performance in detecting HOIs, achieving SOTA results on both V-COCO and HICO-DET datasets, demonstrating the effectiveness of our solution. In future research, we plan to explore the integration of multimodal data, such as combining depth and textual information, to further enhance the accuracy of human–object interaction detection.

## Figures and Tables

**Table 1:** Performance comparison on the V-COCO dataset.

Method	Feature Backbone	Scenario 1	Scenario 2
UnionDet [35]	ResNet-50-FPN	47.5	56.2
Wang et al. [13]	ResNet-50-FPN	51.0	-
PGPN [30]	ResNet-50-FPN	50.2	-
SMPNet [31]	ResNet-50-FPN	52.8	-
HOI-Trans [14]	ResNet-101-FPN	52.9	-
FCMNet [45]	ResNet-50	53.1	-
IDN [46]	ResNet-50	53.3	60.3
ASNet [39]	ResNet-50	53.9	-
GGNet [34]	Hourglass-104	54.7	-
HOTR [17]	ResNet-50	55.2	64.4
GTNet [47]	ResNet-50	56.2	60.1
QPIC [16]	ResNet-50	58.8	61.0
QPIC [16]	ResNet-101	58.3	60.7
Zhang et al. [20]	ResNet-101	60.7	66.2
Liu et al. [8]	ResNet-50	63.0	65.2
Wu et al. [10]	ResNet-50	63.0	65.1
HOICLIP [18]	ResNet-50	63.5	64.8
GEN-VLKT_*l*_ [48]	ResNet-101	63.6	65.9
CDN [19]	ResNet-101	63.9	65.9
OCN [49]	ResNet-50	64.2	66.3
Our method	ResNet-50	63.8	65.5
Our method	ResNet-101	65.2	66.8

**Table 2:** Performance comparison on the HICO-DET dataset.

Method	Full	Rare	Non-Rare
UnionDet [35]	17.58	11.72	19.33
Wang et al. [13]	19.56	12.79	21.58
FCMNet [45]	20.41	17.34	21.56
PGPN [30]	17.40	13.84	18.45
SMPNet [31]	20.31	17.14	21.26
PPDM [12]	21.73	13.78	24.10
HOI-Trans [14]	23.46	16.91	25.41
PST [15]	23.93	14.98	26.60
HOTR [17]	25.10	17.34	27.42
IDN [46]	26.29	22.61	27.39
GTNet [47]	26.78	21.02	28.50
ATL [50]	28.53	21.64	30.59
ASNet [39]	28.87	24.25	30.25
QPIC (ResNet-50) [16]	29.07	21.85	31.23
QPIC (ResNet-101) [16]	29.90	23.92	31.69
GGNet [34]	29.17	22.13	30.84
CDN [19]	32.07	27.19	33.53
Zhang et al. [20]	32.31	28.55	33.44
HOICLIP [19]	34.69	31.12	35.74
Liu et al. [8]	33.51	30.30	34.46
OCN(ResNet-50) [49]	30.91	25.56	32.51
GEN-VLKT_*l*_ [48]	34.95	31.18	36.08
Our method (ResNet-50)	34.47	32.43	33.78
Our method (ResNet-101)	35.06	32.48	35.83

**Table 3:** Ablation studies of PRTT in the V-COCO test set.

Method	${\mathrm{mAP}}_{\mathrm{role}}$
QPIC [16]	58.8
Base model	62.1
Base model + ${\mathrm{PSFE}}_{v}$	63.3
Base model + FR + ${\mathrm{PSFE}}_{v}$	63.6
Base model + FR + ${\mathrm{PSFE}}_{v}$ + IS	64.1
Base model + ${\mathrm{PSFE}}_{s}$ + IS	64.3
Base model + FR + ${\mathrm{PSFE}}_{v}$ + ${\mathrm{PSFE}}_{s}$	64.8
Base model + ${\mathrm{PSFE}}_{v}$ + ${\mathrm{PSFE}}_{s}$ + IS	64.9
Our method (Base model + FR + ${\mathrm{PSFE}}_{v}$ + ${\mathrm{PSFE}}_{s}$ + IS)	65.2

## Data Availability

The authors confirm that the data supporting the findings of this study are available within the article.
